# 2,4-Bis[(3-butyl­imidazol-3-ium-1-yl)meth­yl]-1,3,5-trimethyl­benzene bis­(hexa­fluoro­phosphate)

**DOI:** 10.1107/S1600536811003916

**Published:** 2011-02-05

**Authors:** Rosenani A. Haque, Abbas Washeel Salman, Madhukar Hemamalini, Hoong-Kun Fun

**Affiliations:** a School of Chemical Sciences, Universiti Sains Malaysia, 11800 USM, Penang, Malaysia; b X-ray Crystallography Unit, School of Physics, Universiti Sains Malaysia, 11800 USM, Penang, Malaysia

## Abstract

In the title molecular salt, C_25_H_38_N_4_^2+^·2PF_6_^−^, one of the butyl groups and four F atoms in the basal plane of one of the PF_6_^−^ octa­hedra are disordered over two sets of sites, with occupancy ratios of 0.704 (5):0.296 (5) and 0.71 (3):0.29 (3), respectively. The central benzene ring makes dihedral angles of 85.17 (12) and 81.97 (12)° with the terminal imidazole rings. In the crystal, cations and anions are linked together *via* inter­molecular C—H⋯F hydrogen bonds forming a three-dimensional network.

## Related literature

For applications of *N*-heterocyclic carbenes, see: Tryg *et al.* (2005 ▶); Herrmann (2002 ▶); Tominaga *et al.* (2004 ▶); Magill *et al.* (2001 ▶); Arduengo *et al.* (1991 ▶); Herrmann & Kocher (1997 ▶); Herrmann *et al.* (1998 ▶); McGuinness *et al.* (1999 ▶). For the stability of the temperature controller used in the data collection, see: Cosier & Glazer (1986 ▶).
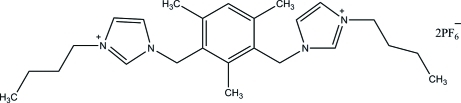


## Experimental

### 

#### Crystal data

C_25_H_38_N_4_^2+^·2PF_6_^−^
*M* *_r_* = 684.53
Monoclinic, 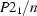

*a* = 12.3851 (2) Å
*b* = 19.6516 (3) Å
*c* = 12.7586 (2) Å
β = 104.698 (1)°
*V* = 3003.66 (8) Å^3^
*Z* = 4
Mo *K*α radiation
μ = 0.24 mm^−1^
*T* = 100 K
0.39 × 0.17 × 0.12 mm

#### Data collection

Bruker SMART APEXII CCD area-detector diffractometer
Absorption correction: multi-scan (*SADABS*; Bruker, 2009 ▶) *T*_min_ = 0.911, *T*_max_ = 0.971
34854 measured reflections
8766 independent reflections
5113 reflections with *I* > 2σ(*I*)
*R*_int_ = 0.064

#### Refinement

*R*[*F* ^2^ > 2σ(*F* ^2^)] = 0.055
*wR*(*F* ^2^) = 0.135
*S* = 1.03
8766 reflections
453 parameters
177 restraints
H-atom parameters constrained
Δρ_max_ = 0.33 e Å^−3^
Δρ_min_ = −0.40 e Å^−3^

### 

Data collection: *APEX2* (Bruker, 2009 ▶); cell refinement: *SAINT* (Bruker, 2009 ▶); data reduction: *SAINT*; program(s) used to solve structure: *SHELXTL* (Sheldrick, 2008 ▶); program(s) used to refine structure: *SHELXTL*; molecular graphics: *SHELXTL*; software used to prepare material for publication: *SHELXTL* and *PLATON* (Spek, 2009 ▶).

## Supplementary Material

Crystal structure: contains datablocks global, I. DOI: 10.1107/S1600536811003916/sj5098sup1.cif

Structure factors: contains datablocks I. DOI: 10.1107/S1600536811003916/sj5098Isup2.hkl

Additional supplementary materials:  crystallographic information; 3D view; checkCIF report

## Figures and Tables

**Table 1:** Hydrogen-bond geometry (Å, °)

*D*—H⋯*A*	*D*—H	H⋯*A*	*D*⋯*A*	*D*—H⋯*A*
C2—H2*A*⋯F5^i^	0.97	2.45	3.312 (3)	149
C5—H5*A*⋯F9*A*^ii^	0.93	2.35	3.235 (9)	159
C6—H6*A*⋯F6^ii^	0.93	2.51	3.248 (3)	136
C6—H6*A*⋯F12^ii^	0.93	2.54	3.145 (3)	123
C7—H7*A*⋯F4^iii^	0.93	2.32	3.140 (3)	146
C15—H15*A*⋯F10*A*^iv^	0.97	2.54	3.103 (9)	117
C15—H15*A*⋯F11^iv^	0.97	2.50	3.353 (3)	147
C19—H19*B*⋯F6^iii^	0.97	2.54	3.327 (3)	138

Symmetry codes: (i) 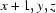; (ii) 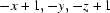; (iii) 
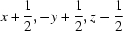
; (iv) 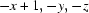.

**Fig. 1.**
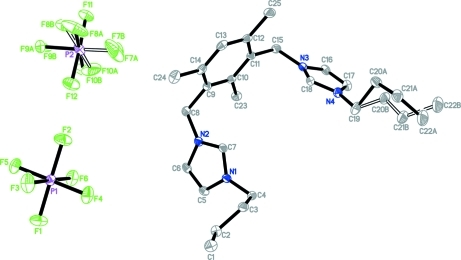
The asymmetric unit of the title compound, showing 30% probability displacement ellipsoids and the atom-numbering scheme. Open bonds represents the minor disorder components [H atoms are omitted for clarity].

**Fig. 2.**
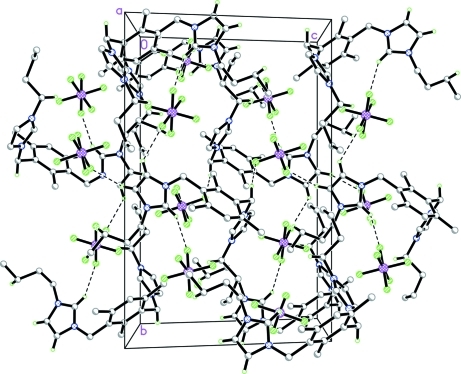
The crystal packing of the title compound, showing a hydrogen-bonded (dashed lines) network. Only atoms of the major disorder components are shown for clarity.

## supplementary crystallographic information

### Comment

*N*-heterocyclic carbenes (NHCs) are now ubiquitous in their usage as ligands for transition metals (Tryg *et al.*, 2005; Herrmann, 2002). These complexes with different metals such as Pd and Ru have been used as catalysts for many reactions; for example C-C coupling reactions and reactions involving olefin metathesis (Tominaga *et al.*, 2004; Magill *et al.*, 2001). This has become an important area of research after the isolation of the first stable crystalline carbene  (Arduengo *et al.*, 1991). NHCs are neutral 2-electron donors, with an ability to bond to both hard and soft metals making them more versatile ligands than phosphines (Herrmann & Kocher, 1997). They are easier to synthesise and functionalise and form stronger bonds to metals leading to more stable metal complexes than metal phosphine complexes (Herrmann *et al.*, 1998). The coordination chemistry of NHCs and their metal complexes continues to be actively studied, particularly for catalytic applications (McGuinness *et al.*, 1999).

The asymmetric unit of the title compound, (Fig. 1),  consists of a 1,3-bis(3-butylimidazolium-1-ylmethyl)mesitylene cation and two hexafluorophosphate anions. One of the butyl groups and four F atoms in the basal plane of one of the PF_6_^-^ octahedra are disordered over two sets of sites, with occupancy ratios of 0.704 (5):0.296 (5) and 0.71 (3):0.29 (3) respectively. The central benzene (C9–C14) ring makes dihedral angles of 85.17 (12)° and 81.97 (12)° with the terminal imidazole (N1/N2/C5–C7)/(N3/N4/C16–C18) rings.

In the crystal structure (Fig. 2), the cations and anions are linked together *via* intermolecular C2—H2A···F5, C5—H5A···F9A, C6—H6A···F6, C6—H6A···F12, C7—H7A···F4, C15—H15A···F10A, C15—H15A···F11 and C19—H19B···F6 (Table 1) hydrogen bonds forming a three-dimensional network.

### Experimental

A mixture of 1,3-bis(bromomethyl)mesitylene (0.9 g, 3.0 mmol) and 1-butylimidazole (0.75 g, 6.0 mmol) in 25 ml of 1,4-dioxane was refluxed at 373 K for 24 h. The resulting slurry was isolated by decantation and washed with fresh 1,4-dioxane (2 x 5 ml) and diethyl ether (2 x 3 ml). The bromide salt was converted directly to its corresponding hexafluorophosphate by a metathesis reaction with methanolic KPF_6_ (1.2 g, 6.5 mmol). The resulting yellowish solid was washed with distilled water and recrystallised from acetonitrile to give pale-yellow crystals. (yield 1.4 g, 88.26 %). Crystals suitable for X-ray diffraction studies were obtained by slow evaporation of the salt solution in acetonitrile at ambient temperature.

### Refinement

All the H atoms were positioned geometrically [ C–H = 0.93–0.97 Å ] and were refined using a riding model, with *U*_iso_(H) = 1.2 or 1.5 *U*_eq_(C). One of the butyl groups and the F7, F8, F9 and F10 fluorine atoms in the one of the phosphate anions are disordered over two sets of sites, with occupancy ratios of 0.704 (5):0.296 (5) and 0.71 (3):0.29 (3) respectively.

### Crystal data

**Table:**

C_25_H_38_N_4_^2+^·2PF_6_^−^	*F*(000) = 1416
*M**_r_* = 684.53	*D*_x_ = 1.514 Mg m^−^^3^
Monoclinic, *P*2_1_/*n*	Mo *K*α radiation, λ = 0.71073 Å
Hall symbol: -P 2yn	Cell parameters from 5155 reflections
*a* = 12.3851 (2) Å	θ = 2.9–27.3°
*b* = 19.6516 (3) Å	µ = 0.24 mm^−^^1^
*c* = 12.7586 (2) Å	*T* = 100 K
β = 104.698 (1)°	Block, pale yellow
*V* = 3003.66 (8) Å^3^	0.39 × 0.17 × 0.12 mm
*Z* = 4	

### Data collection

**Table:**

Bruker SMART APEXII CCD area-detector diffractometer	8766 independent reflections
Radiation source: fine-focus sealed tube	5113 reflections with *I* > 2σ(*I*)
graphite	*R*_int_ = 0.064
φ and ω scans	θ_max_ = 30.1°, θ_min_ = 2.0°
Absorption correction: multi-scan (*SADABS*; Bruker, 2009)	*h* = −17→16
*T*_min_ = 0.911, *T*_max_ = 0.971	*k* = −25→27
34854 measured reflections	*l* = −17→17

### Refinement

**Table:**

Refinement on *F*^2^	Primary atom site location: structure-invariant direct methods
Least-squares matrix: full	Secondary atom site location: difference Fourier map
*R*[*F*^2^ > 2σ(*F*^2^)] = 0.055	Hydrogen site location: inferred from neighbouring sites
*wR*(*F*^2^) = 0.135	H-atom parameters constrained
*S* = 1.03	*w* = 1/[σ^2^(*F*_o_^2^) + (0.051*P*)^2^ + 0.7397*P*] where *P* = (*F*_o_^2^ + 2*F*_c_^2^)/3
8766 reflections	(Δ/σ)_max_ = 0.001
453 parameters	Δρ_max_ = 0.33 e Å^−^^3^
177 restraints	Δρ_min_ = −0.40 e Å^−^^3^

### Special details

**Table:**

Experimental. The crystal was placed in the cold stream of an Oxford Cryosystems Cobra open-flow nitrogen cryostat (Cosier & Glazer, 1986) operating at 100.0 (1) K.
Geometry. All s.u.'s (except the s.u. in the dihedral angle between two l.s. planes) are estimated using the full covariance matrix. The cell s.u.'s are taken into account individually in the estimation of s.u.'s in distances, angles and torsion angles; correlations between s.u.'s in cell parameters are only used when they are defined by crystal symmetry. An approximate (isotropic) treatment of cell s.u.'s is used for estimating s.u.'s involving l.s. planes.
Refinement. Refinement of F^2^ against ALL reflections. The weighted R-factor wR and goodness of fit S are based on F^2^, conventional R-factors R are based on F, with F set to zero for negative F^2^. The threshold expression of F^2^ > 2σ(F^2^) is used only for calculating R-factors(gt) etc. and is not relevant to the choice of reflections for refinement. R-factors based on F^2^ are statistically about twice as large as those based on F, and R- factors based on ALL data will be even larger.

### Fractional atomic coordinates and isotropic or equivalent isotropic displacement parameters (Å^2^)

**Table:**

	*x*	*y*	*z*	*U*_iso_*/*U*_eq_	Occ. (<1)
P1	0.20895 (5)	0.21416 (3)	0.70794 (5)	0.02528 (14)	
F1	0.27007 (13)	0.19236 (8)	0.82874 (11)	0.0516 (4)	
F2	0.15016 (13)	0.23475 (8)	0.58707 (12)	0.0539 (4)	
F3	0.12414 (12)	0.25961 (7)	0.75254 (13)	0.0506 (4)	
F4	0.29111 (12)	0.27811 (7)	0.71659 (12)	0.0457 (4)	
F5	0.12931 (11)	0.14951 (7)	0.69934 (12)	0.0440 (4)	
F6	0.29625 (11)	0.16855 (7)	0.66468 (12)	0.0411 (4)	
P2	0.10834 (6)	0.08585 (3)	0.25608 (5)	0.03360 (16)	
F7A	0.1954 (7)	0.1328 (5)	0.2220 (9)	0.086 (2)	0.71 (3)
F8A	0.0192 (10)	0.1437 (7)	0.2271 (10)	0.095 (3)	0.71 (3)
F9A	0.0210 (6)	0.0378 (6)	0.2895 (7)	0.067 (2)	0.71 (3)
F10A	0.1988 (7)	0.0260 (4)	0.2873 (7)	0.0559 (15)	0.71 (3)
F7B	0.172 (3)	0.1469 (16)	0.231 (3)	0.137 (11)	0.29 (3)
F8B	−0.0055 (14)	0.1272 (12)	0.227 (2)	0.063 (4)	0.29 (3)
F9B	0.0389 (18)	0.0197 (8)	0.2790 (14)	0.060 (4)	0.29 (3)
F10B	0.2128 (15)	0.0415 (15)	0.279 (2)	0.088 (7)	0.29 (3)
F11	0.07227 (13)	0.05889 (8)	0.13307 (11)	0.0469 (4)	
F12	0.14197 (13)	0.11048 (8)	0.37886 (11)	0.0539 (4)	
N1	0.85438 (15)	0.08695 (9)	0.41927 (14)	0.0265 (4)	
N2	0.73557 (15)	0.01726 (9)	0.32089 (14)	0.0269 (4)	
N3	0.84666 (14)	0.11056 (9)	−0.07330 (14)	0.0259 (4)	
N4	0.94852 (15)	0.19703 (9)	−0.00729 (15)	0.0288 (4)	
C1	0.8135 (2)	0.22441 (12)	0.6905 (2)	0.0394 (6)	
H1A	0.7985	0.2044	0.7539	0.059*	
H1B	0.7463	0.2443	0.6467	0.059*	
H1C	0.8695	0.2590	0.7117	0.059*	
C2	0.85494 (19)	0.16985 (12)	0.62574 (19)	0.0333 (5)	
H2A	0.9262	0.1527	0.6679	0.040*	
H2B	0.8025	0.1323	0.6124	0.040*	
C3	0.8684 (2)	0.19657 (12)	0.51876 (18)	0.0344 (6)	
H3A	0.7952	0.2088	0.4743	0.041*	
H3B	0.9126	0.2379	0.5325	0.041*	
C4	0.92215 (19)	0.14853 (12)	0.45473 (18)	0.0322 (5)	
H4A	0.9334	0.1721	0.3915	0.039*	
H4B	0.9948	0.1351	0.4990	0.039*	
C5	0.85966 (19)	0.02770 (11)	0.47726 (18)	0.0306 (5)	
H5A	0.9056	0.0193	0.5459	0.037*	
C6	0.78581 (19)	−0.01605 (11)	0.41627 (18)	0.0301 (5)	
H6A	0.7714	−0.0603	0.4349	0.036*	
C7	0.77865 (18)	0.07964 (11)	0.32502 (17)	0.0272 (5)	
H7A	0.7591	0.1125	0.2711	0.033*	
C8	0.64688 (19)	−0.01183 (11)	0.23136 (18)	0.0307 (5)	
H8A	0.5788	−0.0166	0.2551	0.037*	
H8B	0.6694	−0.0568	0.2139	0.037*	
C9	0.62393 (17)	0.03175 (10)	0.13126 (17)	0.0253 (5)	
C10	0.69484 (17)	0.02784 (10)	0.06159 (17)	0.0247 (5)	
C11	0.67302 (17)	0.06873 (11)	−0.03184 (17)	0.0255 (5)	
C12	0.58185 (18)	0.11365 (11)	−0.05516 (18)	0.0293 (5)	
C13	0.51248 (18)	0.11498 (11)	0.0150 (2)	0.0320 (5)	
H13A	0.4505	0.1435	−0.0012	0.038*	
C14	0.53172 (18)	0.07571 (11)	0.10792 (19)	0.0295 (5)	
C15	0.74935 (18)	0.06444 (12)	−0.10679 (18)	0.0311 (5)	
H15A	0.7756	0.0180	−0.1079	0.037*	
H15B	0.7077	0.0761	−0.1797	0.037*	
C16	0.94168 (18)	0.10753 (12)	−0.11101 (18)	0.0304 (5)	
H16A	0.9588	0.0743	−0.1562	0.036*	
C17	1.0046 (2)	0.16150 (12)	−0.07013 (19)	0.0328 (5)	
H17A	1.0735	0.1728	−0.0820	0.039*	
C18	0.85343 (18)	0.16506 (11)	−0.01042 (17)	0.0266 (5)	
H18A	0.8003	0.1786	0.0254	0.032*	
C19	0.9808 (2)	0.26251 (12)	0.0467 (2)	0.0404 (6)	
H19A	1.0614	0.2662	0.0701	0.048*	0.704 (5)
H19B	0.9509	0.2666	0.1097	0.048*	0.704 (5)
H19C	1.0461	0.2548	0.1047	0.048*	0.296 (5)
H19D	0.9222	0.2762	0.0790	0.048*	0.296 (5)
C20A	0.9304 (4)	0.32135 (17)	−0.0399 (3)	0.0387 (9)	0.704 (5)
H20A	0.9613	0.3165	−0.1022	0.046*	0.704 (5)
H20B	0.8502	0.3157	−0.0646	0.046*	0.704 (5)
C21A	0.9560 (3)	0.39198 (18)	0.0069 (3)	0.0426 (10)	0.704 (5)
H21A	0.9283	0.3962	0.0712	0.051*	0.704 (5)
H21B	0.9173	0.4252	−0.0455	0.051*	0.704 (5)
C22A	1.0797 (4)	0.4072 (3)	0.0360 (5)	0.0587 (13)	0.704 (5)
H22A	1.0920	0.4533	0.0609	0.088*	0.704 (5)
H22B	1.1081	0.4010	−0.0267	0.088*	0.704 (5)
H22C	1.1176	0.3768	0.0924	0.088*	0.704 (5)
C20B	1.0049 (9)	0.3150 (4)	−0.0097 (8)	0.040 (2)	0.296 (5)
H20C	0.9405	0.3245	−0.0693	0.048*	0.296 (5)
H20D	1.0657	0.3019	−0.0407	0.048*	0.296 (5)
C21B	1.0374 (10)	0.3794 (5)	0.0545 (8)	0.047 (3)	0.296 (5)
H21C	1.0987	0.3702	0.1171	0.057*	0.296 (5)
H21D	0.9747	0.3957	0.0801	0.057*	0.296 (5)
C22B	1.0725 (10)	0.4342 (6)	−0.0164 (11)	0.0587 (13)	0.296 (5)
H22D	1.1108	0.4703	0.0289	0.088*	0.296 (5)
H22E	1.0074	0.4522	−0.0665	0.088*	0.296 (5)
H22F	1.1212	0.4144	−0.0558	0.088*	0.296 (5)
C23	0.79482 (18)	−0.01942 (11)	0.08577 (19)	0.0327 (5)	
H23A	0.8134	−0.0317	0.1611	0.049*	
H23B	0.8572	0.0032	0.0693	0.049*	
H23C	0.7772	−0.0597	0.0423	0.049*	
C24	0.4549 (2)	0.08292 (13)	0.1822 (2)	0.0422 (6)	
H24A	0.3953	0.1137	0.1504	0.063*	
H24B	0.4961	0.1004	0.2510	0.063*	
H24C	0.4243	0.0392	0.1923	0.063*	
C25	0.5587 (2)	0.16090 (13)	−0.1517 (2)	0.0424 (6)	
H25A	0.4941	0.1881	−0.1522	0.064*	
H25B	0.5451	0.1346	−0.2172	0.064*	
H25C	0.6219	0.1900	−0.1471	0.064*	

### Atomic displacement parameters (Å^2^)

**Table:**

	*U*^11^	*U*^22^	*U*^33^	*U*^12^	*U*^13^	*U*^23^
P1	0.0270 (3)	0.0194 (3)	0.0286 (3)	−0.0011 (2)	0.0055 (2)	−0.0012 (2)
F1	0.0674 (10)	0.0463 (9)	0.0311 (8)	0.0023 (8)	−0.0061 (7)	−0.0005 (7)
F2	0.0686 (10)	0.0479 (9)	0.0357 (8)	0.0083 (8)	−0.0042 (7)	0.0101 (7)
F3	0.0523 (9)	0.0371 (8)	0.0731 (11)	0.0098 (7)	0.0355 (8)	−0.0018 (8)
F4	0.0525 (9)	0.0246 (7)	0.0672 (11)	−0.0149 (6)	0.0281 (8)	−0.0157 (7)
F5	0.0394 (8)	0.0342 (8)	0.0592 (10)	−0.0139 (6)	0.0142 (7)	−0.0038 (7)
F6	0.0426 (8)	0.0257 (7)	0.0603 (10)	−0.0008 (6)	0.0226 (7)	−0.0104 (7)
P2	0.0460 (4)	0.0291 (3)	0.0283 (3)	0.0019 (3)	0.0144 (3)	0.0021 (3)
F7A	0.105 (4)	0.094 (5)	0.069 (3)	−0.063 (3)	0.041 (3)	−0.002 (3)
F8A	0.166 (8)	0.081 (4)	0.038 (3)	0.087 (5)	0.023 (6)	0.014 (3)
F9A	0.047 (2)	0.114 (6)	0.041 (2)	−0.032 (3)	0.0124 (16)	0.014 (3)
F10A	0.081 (4)	0.054 (3)	0.031 (2)	0.0346 (19)	0.009 (2)	−0.0015 (14)
F7B	0.26 (3)	0.079 (10)	0.073 (9)	−0.093 (14)	0.052 (14)	0.008 (8)
F8B	0.047 (6)	0.107 (13)	0.033 (5)	0.040 (6)	0.010 (3)	0.001 (8)
F9B	0.103 (10)	0.036 (6)	0.025 (5)	−0.021 (5)	−0.014 (6)	0.015 (3)
F10B	0.030 (5)	0.171 (18)	0.057 (9)	0.036 (8)	0.004 (5)	−0.022 (10)
F11	0.0660 (10)	0.0470 (9)	0.0271 (8)	0.0099 (8)	0.0105 (7)	0.0005 (6)
F12	0.0779 (11)	0.0462 (9)	0.0348 (8)	0.0090 (8)	0.0092 (8)	−0.0085 (7)
N1	0.0313 (10)	0.0241 (10)	0.0227 (9)	−0.0024 (7)	0.0045 (7)	0.0013 (7)
N2	0.0347 (10)	0.0199 (9)	0.0258 (9)	−0.0025 (8)	0.0074 (8)	0.0018 (7)
N3	0.0299 (10)	0.0260 (10)	0.0221 (9)	0.0002 (8)	0.0069 (7)	0.0023 (8)
N4	0.0306 (10)	0.0254 (10)	0.0315 (10)	−0.0016 (8)	0.0094 (8)	0.0000 (8)
C1	0.0413 (14)	0.0328 (14)	0.0431 (15)	0.0032 (11)	0.0090 (11)	−0.0040 (11)
C2	0.0339 (13)	0.0310 (13)	0.0361 (13)	0.0022 (10)	0.0110 (10)	0.0014 (10)
C3	0.0428 (14)	0.0264 (12)	0.0294 (12)	−0.0080 (10)	0.0009 (10)	0.0010 (10)
C4	0.0363 (13)	0.0351 (13)	0.0236 (11)	−0.0125 (10)	0.0045 (9)	−0.0017 (10)
C5	0.0367 (12)	0.0273 (12)	0.0269 (12)	0.0054 (10)	0.0065 (9)	0.0063 (9)
C6	0.0430 (13)	0.0198 (11)	0.0293 (12)	0.0030 (9)	0.0125 (10)	0.0061 (9)
C7	0.0339 (12)	0.0236 (11)	0.0231 (11)	−0.0044 (9)	0.0053 (9)	0.0034 (9)
C8	0.0378 (13)	0.0233 (11)	0.0305 (12)	−0.0077 (9)	0.0079 (10)	−0.0031 (9)
C9	0.0294 (11)	0.0184 (10)	0.0267 (11)	−0.0054 (8)	0.0045 (9)	−0.0040 (9)
C10	0.0257 (11)	0.0172 (10)	0.0275 (11)	−0.0017 (8)	−0.0001 (8)	−0.0030 (8)
C11	0.0264 (11)	0.0230 (11)	0.0239 (11)	−0.0038 (8)	0.0006 (8)	−0.0026 (9)
C12	0.0285 (11)	0.0214 (11)	0.0324 (12)	−0.0038 (9)	−0.0026 (9)	−0.0017 (9)
C13	0.0246 (11)	0.0203 (11)	0.0459 (14)	0.0006 (9)	−0.0008 (10)	−0.0069 (10)
C14	0.0269 (11)	0.0219 (11)	0.0390 (13)	−0.0053 (9)	0.0072 (10)	−0.0117 (10)
C15	0.0355 (12)	0.0308 (12)	0.0252 (12)	−0.0059 (10)	0.0044 (9)	−0.0045 (9)
C16	0.0361 (13)	0.0296 (12)	0.0296 (12)	0.0058 (10)	0.0157 (10)	0.0035 (10)
C17	0.0358 (13)	0.0295 (13)	0.0367 (13)	0.0026 (10)	0.0158 (10)	0.0038 (10)
C18	0.0285 (11)	0.0268 (11)	0.0256 (11)	−0.0010 (9)	0.0089 (9)	0.0026 (9)
C19	0.0357 (13)	0.0321 (13)	0.0552 (17)	−0.0076 (11)	0.0152 (12)	−0.0116 (12)
C20A	0.048 (2)	0.033 (2)	0.034 (2)	0.0015 (17)	0.0102 (18)	0.0030 (16)
C21A	0.044 (2)	0.036 (2)	0.049 (2)	0.0031 (17)	0.0127 (19)	0.0075 (17)
C22A	0.059 (3)	0.040 (3)	0.078 (4)	−0.010 (2)	0.020 (3)	0.008 (2)
C20B	0.045 (6)	0.029 (4)	0.045 (5)	−0.009 (4)	0.010 (4)	−0.006 (4)
C21B	0.046 (6)	0.043 (6)	0.053 (6)	−0.015 (5)	0.011 (5)	−0.005 (4)
C22B	0.059 (3)	0.040 (3)	0.078 (4)	−0.010 (2)	0.020 (3)	0.008 (2)
C23	0.0332 (12)	0.0266 (12)	0.0359 (13)	0.0053 (9)	0.0042 (10)	0.0014 (10)
C24	0.0405 (14)	0.0383 (15)	0.0515 (16)	−0.0043 (11)	0.0184 (12)	−0.0166 (12)
C25	0.0394 (14)	0.0377 (14)	0.0423 (15)	−0.0002 (11)	−0.0042 (11)	0.0103 (12)

### Geometric parameters (Å, °)

**Table:**

P1—F2	1.5819 (15)	C10—C11	1.406 (3)
P1—F3	1.5901 (14)	C10—C23	1.516 (3)
P1—F1	1.5937 (14)	C11—C12	1.404 (3)
P1—F5	1.5950 (14)	C11—C15	1.508 (3)
P1—F4	1.6032 (13)	C12—C13	1.389 (3)
P1—F6	1.6063 (14)	C12—C25	1.511 (3)
P2—F7B	1.515 (18)	C13—C14	1.384 (3)
P2—F10B	1.525 (16)	C13—H13A	0.9300
P2—F8A	1.562 (7)	C14—C24	1.510 (3)
P2—F7A	1.562 (6)	C15—H15A	0.9700
P2—F9A	1.575 (6)	C15—H15B	0.9700
P2—F8B	1.587 (15)	C16—C17	1.341 (3)
P2—F12	1.5907 (15)	C16—H16A	0.9300
P2—F10A	1.604 (6)	C17—H17A	0.9300
P2—F11	1.6087 (15)	C18—H18A	0.9300
P2—F9B	1.626 (14)	C19—C20B	1.335 (9)
N1—C7	1.332 (3)	C19—C20A	1.611 (4)
N1—C5	1.372 (3)	C19—H19A	0.9700
N1—C4	1.477 (3)	C19—H19B	0.9700
N2—C7	1.333 (3)	C19—H19C	0.9600
N2—C6	1.383 (3)	C19—H19D	0.9601
N2—C8	1.484 (3)	C20A—C21A	1.512 (5)
N3—C18	1.328 (3)	C20A—H20A	0.9700
N3—C16	1.381 (3)	C20A—H20B	0.9700
N3—C15	1.482 (3)	C21A—C22A	1.512 (5)
N4—C18	1.326 (3)	C21A—H21A	0.9700
N4—C17	1.377 (3)	C21A—H21B	0.9700
N4—C19	1.466 (3)	C22A—H22A	0.9600
C1—C2	1.520 (3)	C22A—H22B	0.9600
C1—H1A	0.9600	C22A—H22C	0.9600
C1—H1B	0.9600	C20B—C21B	1.505 (11)
C1—H1C	0.9600	C20B—H20C	0.9700
C2—C3	1.511 (3)	C20B—H20D	0.9700
C2—H2A	0.9700	C21B—C22B	1.538 (12)
C2—H2B	0.9700	C21B—H21C	0.9700
C3—C4	1.510 (3)	C21B—H21D	0.9700
C3—H3A	0.9700	C22B—H22D	0.9600
C3—H3B	0.9700	C22B—H22E	0.9600
C4—H4A	0.9700	C22B—H22F	0.9600
C4—H4B	0.9700	C23—H23A	0.9600
C5—C6	1.349 (3)	C23—H23B	0.9600
C5—H5A	0.9300	C23—H23C	0.9600
C6—H6A	0.9300	C24—H24A	0.9600
C7—H7A	0.9300	C24—H24B	0.9600
C8—C9	1.504 (3)	C24—H24C	0.9600
C8—H8A	0.9700	C25—H25A	0.9600
C8—H8B	0.9700	C25—H25B	0.9600
C9—C10	1.401 (3)	C25—H25C	0.9600
C9—C14	1.402 (3)		
			
F2—P1—F3	91.31 (9)	C9—C8—H8A	109.2
F2—P1—F1	178.71 (9)	N2—C8—H8B	109.2
F3—P1—F1	89.98 (9)	C9—C8—H8B	109.2
F2—P1—F5	90.75 (8)	H8A—C8—H8B	107.9
F3—P1—F5	91.09 (8)	C10—C9—C14	120.5 (2)
F1—P1—F5	89.20 (8)	C10—C9—C8	119.58 (19)
F2—P1—F4	89.64 (8)	C14—C9—C8	120.0 (2)
F3—P1—F4	90.02 (8)	C9—C10—C11	119.33 (19)
F1—P1—F4	90.38 (8)	C9—C10—C23	121.0 (2)
F5—P1—F4	178.81 (8)	C11—C10—C23	119.6 (2)
F2—P1—F6	89.49 (8)	C12—C11—C10	120.7 (2)
F3—P1—F6	179.03 (9)	C12—C11—C15	120.0 (2)
F1—P1—F6	89.22 (8)	C10—C11—C15	119.28 (19)
F5—P1—F6	89.44 (7)	C13—C12—C11	118.1 (2)
F4—P1—F6	89.44 (7)	C13—C12—C25	119.5 (2)
F7B—P2—F10B	91.8 (11)	C11—C12—C25	122.4 (2)
F7B—P2—F8A	75.5 (12)	C14—C13—C12	122.8 (2)
F10B—P2—F8A	167.3 (10)	C14—C13—H13A	118.6
F10B—P2—F7A	77.3 (12)	C12—C13—H13A	118.6
F8A—P2—F7A	90.0 (5)	C13—C14—C9	118.6 (2)
F7B—P2—F9A	164.5 (13)	C13—C14—C24	119.1 (2)
F10B—P2—F9A	102.2 (10)	C9—C14—C24	122.2 (2)
F8A—P2—F9A	90.5 (4)	N3—C15—C11	112.20 (17)
F7A—P2—F9A	179.3 (6)	N3—C15—H15A	109.2
F7B—P2—F8B	91.5 (12)	C11—C15—H15A	109.2
F10B—P2—F8B	175.6 (12)	N3—C15—H15B	109.2
F7A—P2—F8B	105.5 (9)	C11—C15—H15B	109.2
F9A—P2—F8B	74.9 (9)	H15A—C15—H15B	107.9
F7B—P2—F12	86.9 (13)	C17—C16—N3	107.0 (2)
F10B—P2—F12	89.2 (9)	C17—C16—H16A	126.5
F8A—P2—F12	90.2 (5)	N3—C16—H16A	126.5
F7A—P2—F12	93.9 (4)	C16—C17—N4	107.3 (2)
F9A—P2—F12	86.7 (4)	C16—C17—H17A	126.3
F8B—P2—F12	94.0 (10)	N4—C17—H17A	126.3
F7B—P2—F10A	105.3 (14)	N4—C18—N3	108.6 (2)
F8A—P2—F10A	179.0 (5)	N4—C18—H18A	125.7
F7A—P2—F10A	90.9 (4)	N3—C18—H18A	125.7
F9A—P2—F10A	88.7 (4)	C20B—C19—N4	119.8 (4)
F8B—P2—F10A	163.1 (9)	N4—C19—C20A	107.2 (2)
F12—P2—F10A	89.2 (3)	C20B—C19—H19A	75.8
F7B—P2—F11	95.0 (13)	N4—C19—H19A	110.3
F10B—P2—F11	90.8 (9)	C20A—C19—H19A	110.3
F8A—P2—F11	90.2 (5)	C20B—C19—H19B	124.6
F7A—P2—F11	87.9 (4)	N4—C19—H19B	110.3
F9A—P2—F11	91.5 (4)	C20A—C19—H19B	110.3
F8B—P2—F11	86.0 (10)	H19A—C19—H19B	108.5
F12—P2—F11	178.15 (9)	C20B—C19—H19C	106.1
F10A—P2—F11	90.3 (3)	N4—C19—H19C	107.4
F7B—P2—F9B	178.3 (14)	C20A—C19—H19C	138.2
F10B—P2—F9B	88.2 (10)	H19B—C19—H19C	78.4
F8A—P2—F9B	104.5 (7)	C20B—C19—H19D	108.2
F7A—P2—F9B	163.0 (9)	N4—C19—H19D	107.7
F8B—P2—F9B	88.4 (9)	C20A—C19—H19D	83.7
F12—P2—F9B	94.9 (7)	H19A—C19—H19D	132.7
F10A—P2—F9B	74.7 (8)	H19C—C19—H19D	107.1
F11—P2—F9B	83.3 (7)	C21A—C20A—C19	112.5 (3)
C7—N1—C5	108.76 (18)	C21A—C20A—H20A	109.1
C7—N1—C4	125.47 (18)	C19—C20A—H20A	109.1
C5—N1—C4	125.77 (18)	C21A—C20A—H20B	109.1
C7—N2—C6	108.33 (18)	C19—C20A—H20B	109.1
C7—N2—C8	126.56 (18)	H20A—C20A—H20B	107.8
C6—N2—C8	125.10 (17)	C20A—C21A—C22A	112.1 (4)
C18—N3—C16	108.45 (19)	C20A—C21A—H21A	109.2
C18—N3—C15	126.12 (19)	C22A—C21A—H21A	109.2
C16—N3—C15	125.17 (19)	C20A—C21A—H21B	109.2
C18—N4—C17	108.53 (19)	C22A—C21A—H21B	109.2
C18—N4—C19	124.3 (2)	H21A—C21A—H21B	107.9
C17—N4—C19	126.94 (19)	C19—C20B—C21B	114.9 (8)
C2—C1—H1A	109.5	C19—C20B—H20C	108.5
C2—C1—H1B	109.5	C21B—C20B—H20C	108.5
H1A—C1—H1B	109.5	C19—C20B—H20D	108.5
C2—C1—H1C	109.5	C21B—C20B—H20D	108.5
H1A—C1—H1C	109.5	H20C—C20B—H20D	107.5
H1B—C1—H1C	109.5	C20B—C21B—C22B	110.2 (9)
C3—C2—C1	112.05 (19)	C20B—C21B—H21C	109.6
C3—C2—H2A	109.2	C22B—C21B—H21C	109.6
C1—C2—H2A	109.2	C20B—C21B—H21D	109.6
C3—C2—H2B	109.2	C22B—C21B—H21D	109.6
C1—C2—H2B	109.2	H21C—C21B—H21D	108.1
H2A—C2—H2B	107.9	C21B—C22B—H22D	109.5
C4—C3—C2	115.88 (19)	C21B—C22B—H22E	109.5
C4—C3—H3A	108.3	H22D—C22B—H22E	109.5
C2—C3—H3A	108.3	C21B—C22B—H22F	109.5
C4—C3—H3B	108.3	H22D—C22B—H22F	109.5
C2—C3—H3B	108.3	H22E—C22B—H22F	109.5
H3A—C3—H3B	107.4	C10—C23—H23A	109.5
N1—C4—C3	112.56 (18)	C10—C23—H23B	109.5
N1—C4—H4A	109.1	H23A—C23—H23B	109.5
C3—C4—H4A	109.1	C10—C23—H23C	109.5
N1—C4—H4B	109.1	H23A—C23—H23C	109.5
C3—C4—H4B	109.1	H23B—C23—H23C	109.5
H4A—C4—H4B	107.8	C14—C24—H24A	109.5
C6—C5—N1	107.29 (19)	C14—C24—H24B	109.5
C6—C5—H5A	126.4	H24A—C24—H24B	109.5
N1—C5—H5A	126.4	C14—C24—H24C	109.5
C5—C6—N2	107.08 (19)	H24A—C24—H24C	109.5
C5—C6—H6A	126.5	H24B—C24—H24C	109.5
N2—C6—H6A	126.5	C12—C25—H25A	109.5
N1—C7—N2	108.55 (19)	C12—C25—H25B	109.5
N1—C7—H7A	125.7	H25A—C25—H25B	109.5
N2—C7—H7A	125.7	C12—C25—H25C	109.5
N2—C8—C9	112.20 (17)	H25A—C25—H25C	109.5
N2—C8—H8A	109.2	H25B—C25—H25C	109.5
			
C1—C2—C3—C4	172.95 (19)	C25—C12—C13—C14	−176.7 (2)
C7—N1—C4—C3	92.1 (3)	C12—C13—C14—C9	−1.3 (3)
C5—N1—C4—C3	−88.6 (3)	C12—C13—C14—C24	177.1 (2)
C2—C3—C4—N1	64.3 (3)	C10—C9—C14—C13	0.0 (3)
C7—N1—C5—C6	0.2 (3)	C8—C9—C14—C13	−179.73 (18)
C4—N1—C5—C6	−179.2 (2)	C10—C9—C14—C24	−178.4 (2)
N1—C5—C6—N2	−0.1 (3)	C8—C9—C14—C24	1.9 (3)
C7—N2—C6—C5	0.0 (2)	C18—N3—C15—C11	−21.2 (3)
C8—N2—C6—C5	−178.6 (2)	C16—N3—C15—C11	165.40 (19)
C5—N1—C7—N2	−0.2 (3)	C12—C11—C15—N3	92.8 (2)
C4—N1—C7—N2	179.2 (2)	C10—C11—C15—N3	−86.7 (2)
C6—N2—C7—N1	0.1 (2)	C18—N3—C16—C17	−0.5 (2)
C8—N2—C7—N1	178.71 (19)	C15—N3—C16—C17	173.93 (19)
C7—N2—C8—C9	11.8 (3)	N3—C16—C17—N4	0.3 (2)
C6—N2—C8—C9	−169.8 (2)	C18—N4—C17—C16	−0.1 (2)
N2—C8—C9—C10	80.2 (2)	C19—N4—C17—C16	−174.8 (2)
N2—C8—C9—C14	−100.1 (2)	C17—N4—C18—N3	−0.2 (2)
C14—C9—C10—C11	0.3 (3)	C19—N4—C18—N3	174.7 (2)
C8—C9—C10—C11	−179.96 (18)	C16—N3—C18—N4	0.4 (2)
C14—C9—C10—C23	179.98 (18)	C15—N3—C18—N4	−173.91 (18)
C8—C9—C10—C23	−0.3 (3)	C18—N4—C19—C20B	−122.8 (6)
C9—C10—C11—C12	0.6 (3)	C17—N4—C19—C20B	51.1 (7)
C23—C10—C11—C12	−179.06 (19)	C18—N4—C19—C20A	−87.6 (3)
C9—C10—C11—C15	−179.96 (18)	C17—N4—C19—C20A	86.3 (3)
C23—C10—C11—C15	0.4 (3)	C20B—C19—C20A—C21A	−63.4 (7)
C10—C11—C12—C13	−1.8 (3)	N4—C19—C20A—C21A	178.8 (3)
C15—C11—C12—C13	178.77 (19)	C19—C20A—C21A—C22A	65.0 (5)
C10—C11—C12—C25	177.0 (2)	N4—C19—C20B—C21B	−179.7 (7)
C15—C11—C12—C25	−2.4 (3)	C20A—C19—C20B—C21B	103.6 (12)
C11—C12—C13—C14	2.2 (3)	C19—C20B—C21B—C22B	175.3 (9)

### Hydrogen-bond geometry (Å, °)

**Table:**

*D*—H···*A*	*D*—H	H···*A*	*D*···*A*	*D*—H···*A*
C2—H2A···F5^i^	0.97	2.45	3.312 (3)	149
C5—H5A···F9A^ii^	0.93	2.35	3.235 (9)	159
C6—H6A···F6^ii^	0.93	2.51	3.248 (3)	136
C6—H6A···F12^ii^	0.93	2.54	3.145 (3)	123
C7—H7A···F4^iii^	0.93	2.32	3.140 (3)	146.
C15—H15A···F10A^iv^	0.97	2.54	3.103 (9)	117
C15—H15A···F11^iv^	0.97	2.50	3.353 (3)	147
C19—H19B···F6^iii^	0.97	2.54	3.327 (3)	138

Symmetry codes: (i) *x*+1, *y*, *z*; (ii) −*x*+1, −*y*, −*z*+1; (iii) *x*+1/2, −*y*+1/2, *z*−1/2; (iv) −*x*+1, −*y*, −*z*.
